# Bullying and Victimization Trajectories in the First Years of Secondary Education: Implications for Status and Affection

**DOI:** 10.1007/s10964-020-01385-w

**Published:** 2021-01-19

**Authors:** Elsje de Vries, Tessa M. L. Kaufman, René Veenstra, Lydia Laninga-Wijnen, Gijs Huitsing

**Affiliations:** Department of Sociology, University of Groningen, Groningen, the Netherlands

**Keywords:** Social position, Bullying and victimization trajectories, Adolescence, Secondary education

## Abstract

Bullying is known to be associated with social status, but it remains unclear how bullying involvement over time relates to social position (status and affection), especially in the first years at a new school. The aim of this study was to investigate whether (the development of) bullying and victimization was related to the attainment of status (perceived popularity) and affection (friendships, acceptance, rejection) in the first years of secondary education (six waves). Using longitudinal data spanning the first- and second year of secondary education of 824 adolescents (51.5% girls; *M*_*ag*e_ T1 = 12.54, *SD* = 0.45) in the SNARE-study, joint bullying and victimization trajectories were estimated using parallel Latent Class Growth Analysis (LCGA). The four trajectories (decreasing bully, stable high bully, decreasing victim, uninvolved) were related to adolescents’ social position using multigroup analysis that examined differences in slope and intercepts (T1 and T6) of social positions, and indicated that the relative social position of the different joint trajectories was determined at the start of secondary education and did not change over time, with one exception: adolescents continuing bullying were besides being popular also increasingly rejected over time. Although bullying is functional behavior that serves to optimize adolescents’ social position, anti-bullying interventions may account for the increasing lack of affection that may hinder bullies’ long-term social development.

## Introduction

In early adolescence, youths’ focal goal is to achieve a strong social position in the peer group. In the first place because adolescents experience important social, cognitive, and behavioral changes (Viau et al. 2020) that make them increasingly susceptible to their peers’ perceptions about them and with “fitting in” (Veenstra and Laninga-Wijnen 2021). Moreover, in many countries including the Netherlands, these developmental changes coincide with the transition from smaller primary schools to larger secondary schools around the ages of 12-14. The school transition is causing destabilization and re-organization of adolescents’ social landscape. Primary school friends might go to different secondary schools, and the selected secondary school is often located outside the trusted environment. Thus, adolescents have to re-establish their social position in the new peer context, during a period when peer relations are already highly valued (Laursen and Veenstra 2021). A strong social position provides them with access to resources, including having friends and being treated respectfully by peers (Reijntjes et al. 2013). A seemingly easy, yet harmful way to achieve or solidify a strong social position is by bullying others (Pouwels et al. 2018). However, it remains unknown whether involvement in bullying relates to adolescents’ status (perceived popularity) and affection (friendships, acceptance) in the first years at a new school. Second, most knowledge on bullying in relation to social position has focused on bullying involvement in one moment of time. Therefore, it is unknown how bullying involvement over time relates to social position. For example, does the potential effect of bullying in terms of social position last for a longer period, or do adolescents need to continue their behavior in order to maintain their position? These are important questions because for our understanding of the social dynamics in the first years of secondary education, which may be vital for the content of anti-bullying interventions. Addressing these questions, the key aim of our study is to examine whether bullying and victimization processes are related to adolescents’ social position in the first years of secondary education.

### Social Position in Adolescence and Associated Bullying Involvement

The need for a strong social position is explained by the Social Production Function (SPF) theory (Ormel et al. 1999), which states that individuals seek to optimize both their physical and their social well-being, by five important goals (stimulation, comfort, behavioral confirmation, status and affection). Although all goals are important, some gain extra attention at specific age periods, such as social well-being does in adolescence. Social well-being is achieved by optimizing status and affection. Both status (vertical relationships, perceived popularity) and affection (horizontal relationships, such as friendships, being accepted, and avoidance of being rejected) are essential for the fulfillment of a strong social position and have therefore an important role in steering behavior (Sijtsema et al. 2020).

The attainment of social position may be strongly related to involvement in bullying: as a bully, a victim, a bully-victim or not at all. Bullying is an important type of interpersonal aggression that is often strategically used to fulfill status goals (Sijtsema et al. 2020). Through bullying, adolescents aim to acquire social power over others (Olthof et al. 2011). Their behavior is accepted by most classmates; during adolescence, relative to (early) childhood or adulthood, bullying is viewed less negatively (Pellegrini and Long 2002). Popular, aggressive (tough) peers are seen as ‘cool’ by their peers and are at low risk to become victimized (Rodkin et al. 2006). Nevertheless, bullying may come at the price of other social goals: through bullying, bullies may prioritize vertical relationships, such as status, over horizontal relationships, such as affection (Nocentini et al. 2013). When bullying backfires, it can lead to a loss of affection (e.g., Garandeau and Lansu 2019). Despite the knowledge that bullying relates to status and affection, it is unknown how this behavior over time relates to social position. Do bullies need to continue their bullying behavior in order to remain popular, or is bullying at the start of secondary education, when the hierarchy is determined, enough to remain popular for a prolonged period of time?

Some adolescents refrain from bullying as a means to attain status because they do not want to jeopardize the realization of affection (Sijtsema et al. 2020). These adolescents probably value horizontal relationships, such as friendships, over vertical relationships, such as popularity. They behave in other, more prosocial ways to promote friendships. Thus, it can be expected that adolescents who refrain from bullying have a lower status than bullies, but receive more affection (friends) over time.

Often at the bottom of the hierarchy are the adolescents who are the targets of bullying, and are the lowest in both attained status and affection. However, victims’ status and affection *before* they became victimized is difficult to be determined. On the one hand, bullies may choose victims who are already disliked or perceived as unpopular by their peers (e.g., Veenstra et al. 2010), because those victims are rarely defended by peers and bullies do not face the risk of losing affection. These victims are therefore ‘easy targets’. On the other hand, peers with relatively high status are also likely to become victimized because they are the direct ‘rivals’ of bullies that want to attain status (Faris and Felmlee 2014). Targeting these ‘rivals’ may be most beneficial for attaining a strong social position (Andrews et al. 2016). Finally, it is known that high status bullies often switch between different victims in order to maintain their status (van der Ploeg et al. 2020). However, regardless of their initial status, victims may decrease in status and affection because peers may avoid to form relationships with victims because they may fear that associating with low-status peers would lead to a decrease in their own social status, placing them at a higher risk for victimization in the future (Neal and Veenstra 2021). Especially persistent victims, who are bullied over a prolonged time by multiple bullies became less accepted, more rejected and perceived as less popular by their classmates (Van der Ploeg et al. 2015).

The relation between bullying or victimization and social position has often been examined through cross-sectional or single-assessment data and often in isolation from each other (Zych et al. 2018). However, bullying and victimization are theoretically and empirically entwined, and (the combination of) bullying and victimization behavior within adolescents can change over time. Therefore, the information on the impact of bullying and victimization behavior for social positions in the first years of secondary education can only be achieved through a longitudinal design that takes into account that bullying and victimization rarely happen in isolation (Zych et al. 2018). Moreover, cross-sectional data make it impossible to understand whether bullying needs to continue in order to retain one’s status, or whether it is already functional in the beginning of the process of establishing the peer hierarchy and can then be stopped in order to retain affection. The few longitudinal studies on bullying and victimization indicated that bullying and victimization peaks at the start of secondary education (Pellegrini and Long 2002), which coincides with the general peak in bullying behavior during middle school age (eleven to thirteen years old). After the initial peak, bullying and victimization are likely to remain stable or decrease as children mature (e.g., Haltigan and Vaillancourt 2014), but this may not apply to all forms of bullying, with direct forms decreasing, whereas indirect relational forms of bullying might persist (e.g., Scheithauer et al. 2006).

## Current study

Bullying is motivated by the need for a strong social position, however it is yet unknown if and how bullies in the end succeed. Therefore, the aim of the current study was to investigate whether (the development of) bullying and victimization was related to the attainment of status and affection in the first years of secondary education. Using longitudinal data spanning the first- and second year of secondary education, joint bullying and victimization trajectories were estimated and were related to adolescents’ social position development. It was expected to find trajectories for adolescents involved in bullying, victimization, both, and uninvolved trajectories, which would be stable or decreasing over time. It was expected that stable levels of bullying would be related to the attainment of status (Hypothesis 1a), but not necessarily to the attainment of affection (Hypothesis 1b). Second, it was expected that victimization would be related to lower status (Hypothesis 2a) and lower affection (Hypothesis 2b) over time, independent of social status and affection and T1. Third, it was expected that remaining uninvolved as both a bully and a victim would be related to the attainment of affection (Hypothesis 3a), but not necessarily to the attainment of status (Hypothesis 3b). When data indicates that bullying is indeed functional behavior in order to optimize adolescents’ social position, developers of anti-bullying interventions need to take that function into account.

## Method

### Procedure

The data used in this study stem from the Social Network Analysis of Risk behavior in Early adolescence (SNARE) study, a prospective cohort study on the social development of early adolescents, conducted in two secondary schools in the middle and north of the Netherlands (Franken et al. 2017). Participants were recruited in their first or second grade of school (cohort 1, school year 2011-2012). After a year, a second cohort was added, including students in their first grade at the same schools (cohort 2, school year 2012-2013). Participants received an information letter for themselves and their parents. A passive consent procedure was used. If students did not want to participate, or their parents disagreed with their children’s participation, they were asked to send a reply card or email within two weeks. Students were allowed to opt out participation any time.

In September 2011 (September 2012 for cohort 2), when participating students started secondary school, there was a pre-assessment (T0). At T0, mainly demographic and psychological factors were assessed. No peer reports on bullying, victimization and social position were assessed yet. Subsequently, there were six regular measurement waves in October (T1 and T4), December (T2 and T5) and March (T3 and T6) over two years. Cohort 1 started the regular measurement waves in October 2011 and cohort 2 started the regular measurement waves in October 2012. During the six waves, peer reports on bullying, victimization and social position were assessed. During the assessments, a teacher and research assistant were present to introduce the assessment and to help set up the questionnaires. Students had to fill in an online questionnaire with both self-report and peer nomination questions. The assessment was during school hours and lasted approximately 45 min.

### Participants

In total, 1,826 students were asked to participate in the SNARE study of which 40 students (2.2%) refused to participate (Appendix 1). For the present study, students from cohort 1 and 2 for whom T1 was at the start of their first year of secondary school were eligible for the study. Also, only students that participated for two years in the study were included. This resulted in a sample of 824 participants (*N* cohort 1 = 554; *N* cohort 2 = 270) adolescents (51.5% girls) at T1 (*M*_*ag*e_ = 12.54, *SD* = 0.45). Of the students that participated in the study and were eligible, no one was excluded and Full Information Maximum Likelihood (FIML) methodology of M*plus* was used to estimate missing answers.

### Measures

All data came from peer nominations. Students could nominate an unlimited number of classmates per question by clicking on the students’ names.

#### Bullying and victimization

Victimization (T1—T6) was assessed with received peer nominations on the item ‘Who do you bully?’, and bullying (T1—T6) was assessed with received peer nominations on the item ‘Who bullies you?’. The nominations received were summed to determine bullying and victimization per wave (see Table 1 for the received nominations).

**Table 1:** Number of incoming victim and bully nominations per time point

	Victim (incoming nominations)						Bully (incoming nominations)					
	T1	T2	T3	T4	T5	T6	T1	T2	T3	T4	T5	T6
*M*	0.23	0.17	0.17	0.22	0.22	0.23	0.31	0.36	0.47	0.33	0.25	0.42
0	686 (83.3%)	716 (86.9%)	721 (87.5%)	700 (85.0%)	691 (83.9%)	645 (78.3%)	641 (77.8%)	627 (76.1%)	556 (67.5%)	626 (76.0%)	661 (80.2%)	556 (67.5%)
1	112 (13.6%)	81 (9.8%)	79 (9.6%)	81 (9.8%)	101 (12.3%)	150 (18.2%)	126 (15.3%)	142 (17.2%)	185 (22.5%)	142 (17.2%)	128 (15.5%)	182 (22.1%)
2	11 (1.3%)	21 (2.5%)	15 (1.8%)	35 (4.2%)	21 (2.6%)	15 (1.8%)	46 (5.6%)	34 (4.1%)	58 (7.0%)	46 (5.6%)	26 (3.2%)	68 (8.3%)
>2	15 (1.8%)	6 (0.7%)	9 (1.0%)	8 (1.0%)	9 (1.1%)	3 (0.3%)	11 (1.3%)	21 (2.5%)	25 (3.0%)	10 (1.2%)	7 (0.8%)	7 (0.8%)

*T* time point, *M* mean per time point

#### Social positions

Social positions were measured with perceived popularity (as an indicator for *social status*) and acceptance, rejection, and friendships (as indicators for *affection*) (Sijtsema et al. 2020). Perceived popularity (T1—T6) was assessed with two items: ‘Who are the most popular?’ and ‘Who are the least popular?’ The final score, on a continuous scale, was derived from subtracting the number of least popular nominations from the most popular nominations (Cillessen and Rose 2005).

Acceptance, rejection and friendships (T1—T6) were assessed with: ‘Who do you like (who is nice)?’, ‘Which classmates do you dislike?’ and ‘Which classmates are your best friends?’, respectively. These items were separately used in the analysis.

### Statistical Analyses

First, Latent Class Growth Analysis (LCGA) in M*plus* version 8.4 (Muthén and Muthén 2015) was used to estimate *separate* bullying and victimization trajectories using six time points from T1 to T6, that describe the course of these variables over time. The most optimal solution was tested for the number of trajectories separately for bullying and victimization. A series of multilevel models with nesting at T1 were fitted for both bullying and victimization separately, going from a one-class solution to a six-class solution at maximum (Barker et al. 2008). To choose the optimal model for separate bullying or victimization trajectories, the models were compared using (a) the Akaike Information Criterion (AIC; lowest value), (b) the Bayesian Information Criterion (BIC; lowest value), (c) the adjusted Bayesian Information Criterion (aBIC; lowest value), (d) the Lo–Mendell–Rubin Test (LMRT; significant *p*-values indicate that a model with an additional class is a better fit than a model with one less class), (e) the Vuong–Lo–Mendell–Rubin likelihood ratio test (VLMRT; significant *p*-values indicate that a model with an additional class is a better fit than a model with one less class), (f) the Bootstrapped Likelihood Ratio Test (BLRT; significant *p*-values indicate that a model with an additional class is a better fit than a model with one less class), (g) the entropy (values close to 1 indicates a good classification) and (h) the theoretical meaningfulness.

Second, a parallel process LGCA in M*plus* version 8.4 was used to estimate *joint* bullying and victimization trajectories, assigning individuals a single class membership to both bully and victimization trajectories. Based on the separate LGCA of bullying and victimization, a fixed number of four possible joint bullying and victimization trajectories was estimated. Because of the fixed number of trajectories, no models were compared for a better fit. This joint model was used, because bullying and victimization are theoretically and empirically entwined. The joint bullying and victimization trajectories were useful because they capture the developmental overlap between the distinct, but related, phenomena.

Third, multigroup analysis were used to examine differences in slope and intercepts (T1 and T6) of social positions between the trajectory classes. With the T1 data it could be determined whether perceived popularity, acceptance, rejection, and friendships differed between groups directly at the start of secondary education. With the data at T6, it could be determined whether belonging to a certain bullying and victimization trajectory has led to the attainment of perceived popularity, acceptance, or friendships and avoidance of rejection after the first two years of secondary education. With the slope analysis it could be determined whether the trajectories differed in the development of social positions. Differences between trajectories on these factors were based on their 95% confidence intervals (*CI*’s) (Pfister and Janczyk 2013). In all models, results were adjusted for sex. Missing data was handled using FIML estimation. Finally, to take the dependencies in the data into account, a multilevel structure was used with the cluster (classroom identifier) command in M*plus*.

## Results

### Bullying and Victimization Trajectories

Table 2 displays the model fit indices for the estimation of *victimization* trajectories with Latent Growth Class Analysis (LGCA). The two-class model appeared to be the optimal model for victimization (entropy = 0.991). All fit indicators indicated that the two-class model was better than the one-class model, although the (V)LMRT (*p* = 0.09) and the VLMRT (*p* = 0.09) were only marginally significant. However, theoretically it would not make sense to retain the one-class model. This would mean that there is almost no victimization (*I* = 0.21, *S* = -0.03), which is also in contrast to the findings from Table 1, with at least 103 adolescents (12.5%, at T3) who were nominated as victims. Table 3 displays the model fit indices for the estimation of *bullying* trajectories with LGCA. Here, also the two-class model appeared to be the optimal model for bullying (entropy = 0.976). All fit indicators indicated that the two-class model was better than the one-class model. Thus, for both bullying and victimization, a two-class model was the optimal model. Hence, in the parallel process LGCA, to estimate joint bullying and victimization trajectories, two bully groups and two victimization groups were included which resulted in a four-class joint bullying and victimization model.

**Table 2:** Model fit indices for victimization: one to six latent classes

Victimization													
	1	2	3	4	5	6	AIC	BIC	aBIC	Entropy	LMRT	VLMRT	BLRT
1	N = 824 I = 0.21** S = −0.03* Q = 0.01*						8172.1	8214.5	8185.9				
2	N = 32 I = 2.01** S = −0.22 Q = 0.00	N = 792 I = 0.14** S = −0.03 Q = 0.01**					7179.8	7241.1	7199.8	0.991	0.09	0.09	<0.001
3	N = 19 I = 0.67* S = 0.84** Q = −0.17*	N = 787 I = 0.14** S = −0.03* Q = 0.01**	N = 18 I = 3.30** S = −1.50** Q = 0.21**				6900.8	6981.0	6927.0	0.993	0.38	0.37	<0.001
4	N = 16 I = 2.02** S = 0.24 Q = −0.12*	N = 641 I = 0.09** S = 0.06** Q = −0.02**	N = 18 I = 1.07** S = −0.68** Q = 0.19**	N = 149 I = 0.45** S = −0.35** Q = 0.09**			5908.6	6007.6	5940.9	0.995	0.15	0.15	<0.001
5	N = 15 I = 0.67** S = −0.52** Q = 0.16**	N = 638 I = 0.09** S = 0.06** Q = −0.01**	N = 3 I = 3.13** S = −1.53 Q = 0.33	N = 151 I = 0.51** S = −0.37** Q = 0.01**	N = 17 I = 1.48** S = 0.55 Q = −0.17**		3820.5	3938.4	3859.0	0.995	0.77	0.77	<0.001
6	N = 142 I = 0.35** S = −0.31** Q = 0.09**	N = 639 I = 0.09** S = 0.05** Q = −0.01**	N = 3 I = 3.21** S = −1.62 Q = 0.34	N = 16 I = 1.58* S = 0.47 Q = −0.16	N = 9 I = 2.75* S = −1.11 Q = 0.15	N = 15 I = 0.65** S = −0.50** Q = 0.15**	3539.7	3676.4	3585.4	0.994	0.83	0.83	<0.001

*AIC* Akaike Information Criterion, *BIC* Bayesian Information Criterion, *aBIC* adjusted Bayesian Information Criterion, *LMRT* Lo–Mendell–Rubin Test, *VLMRT* Vuong–Lo–Mendell–Rubin likelihood ratio test, *BLRT* Bootstrapped Likelihood Ratio Test, *I* intercept, *S* slope, *Q* quadratic slope

*p < 0.05; **p < 0.01

**Table 3:** Model fit indices for bullying: one to six latent classes

Bullying													
	1	2	3	4	5	6	AIC	BIC	aBIC	Entropy	LMRT	VLMRT	BLRT
1	N = 824 I = 0.34** S = −0.01 Q = 0.00						10542.6	10585.0	10556.5				
2	N = 763 I = 0.22** S = 0.00 Q = 0.00	N = 61 I = 1.80** S = −0.05 Q = −0.01					9471.2	9532.5	9491.2	0.976	0.01	0.01	<0.001
3	N = 54 I = 1.97** S = −0.77** Q = 0.10**	N = 730 I = 0.14** S = 0.04* Q = 0.00	N = 40 I = 1.40** S = 0.41** Q = −0.08**				9160.2	9240.3	9186.3	0.969	0.17	0.16	<0.001
4	N = 641 I = 0.00 S = 0.14** Q = −0,02**	N = 126 I = 1.00** S = −0.23** Q = 0.03**	N = 46 I = 2.00** S = −0.60** Q = 0.07**	N = 11 I = 3.45** S = −1.29** Q = 0.18**			7621.2	7720.2	7653.5	1.000	0.81	0.81	<0.001
5	N = 36 I = 2.02** S = −0.88** Q = 0.09**	N = 129 I = 0.58** S = 0.19** Q = −0.02	N = 7 I = 0.99* S = 0.79** Q = -0.06	N = 629 I = 0.18** S = −0.06** Q = 0.00	N = 26 I = 0.76** S = 0.54** Q = −0.06*		6598.9	6716.8	6637.4	0.985	1.00	1.00	<0.001
6	N = 641 I = 0.00 S = 0.13** Q = −0.02**	N = 4 I = 4.25** S = −1.71** Q = 0.21**	N = 20 I = 1.00** S = 0.53** Q = -0.08*	N = 7 I = 3.00** S = −1.06** Q = 0.16**	N = 106 I = 1.00** S = −0.37** Q = 0.05**	N = 46 I = 2.00** S = −0.61** Q = 0.07**	5868.4	6005.1	5913.0	0.998	1.00	1.00	<0.001

*AIC* Akaike Information Criterion, *BIC* Bayesian Information Criterion, *aBIC* adjusted Bayesian Information Criterion, *LMRT* Lo–Mendell–Rubin Test, *VLMRT* Vuong–Lo–Mendell–Rubin likelihood ratio test, *BLRT* Bootstrapped Likelihood Ration Test, *I* intercept, *S* slope, *Q* quadratic slope

*p < 0.05; **p < 0.01

### Joint Bullying and Victimization Trajectories

**Fig. 1:**
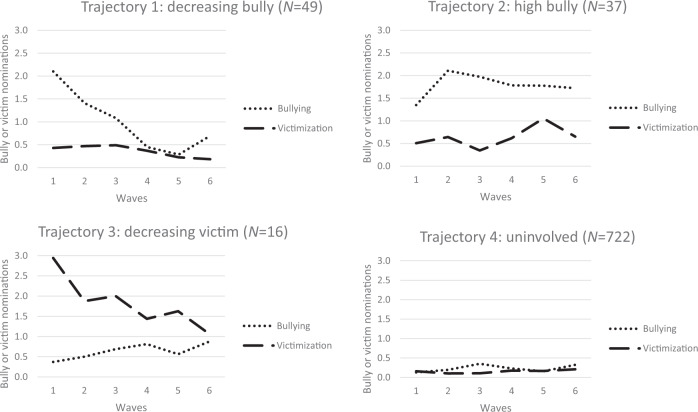
Estimated joint bullying and victimization trajectories

Table 4 shows the parallel process LGCA results of the joint bullying and victimization trajectories. Based on the single LGCA of bullying and victimization, a fixed number of four possible joint trajectories was estimated. Figure 1 displays four joint bullying and victimization trajectories, each trajectory in a separate graph.

**Table 4:** Model fit indices for combined bullying and victimization trajectories: four class solution

	Combined bullying and victimization trajectories							
	Trajectory 1: Decreasing bully (*N* = 49)	Trajectory 2: High bully (*N* = 37)	Trajectory 3: Decreasing victim (*N* = 16)	Trajectory 4: Uninvolved (*N* = 722)	AIC 16454.8	BIC 16638.6	aBIC 16514.8	Entropy 0.984
Bullying	I = 2.11** S = −0.87** Q = 0.11**	I = 1.41** S = 0.37* Q = −0.07**	I = 0.39* S = 0.14 Q = −0.01	I = 0.15** S = 0.04* Q = 0.00				
Victimization	I = 0.43** S = 0.04 Q = −0.02	I = 0.31** S = −0.04 Q = 0.02	I = 2.69** S = −0.51 Q = 0.04	I = 0.14** S = −0.03* Q = 0.01**				

*AIC* Akaike Information Criterion, *BIC* Bayesian Information Criterion, *aBIC* adjusted Bayesian Information Criterion, *I* intercept, *S* slope, *Q* quadratic slope

*p < 0.05; **p < 0.01

The first joint trajectory (*N* = 49), labeled as the *decreasing bully* trajectory, represents adolescents who initially bullied a lot (*M*_intercept_ = 2.11), but this decreased over time *(M*_slope_ = −0.87*; M*_qslope_ = 0.11). At the same time, these adolescents were rarely involved in victimization (*M*_intercept_ = 0.43; *M*_slope_ = 0.04*; M*_qslope_ = −0.02). The second joint trajectory (*N* = 37), labeled as the *high bully* group, represents adolescents who were nominated as a bully during the entire measurement period (*M*_intercept_ = 1.41*; M*_slope_ = 0.37*; M*_qslope_ = −0.07) and only sometimes as a victim (*M*_intercept_ = 0.31*; M*_slope_ = −0.04*; M*_qslope_ = 0.02). The third joint trajectory (*N* = 16), labeled as the *decreasing victim* trajectory, represents adolescents who were initially victimized a lot (*M*_intercept_ = 2.69), but this decreased over time (*M*_slope_ = −0.51; *M*_qslope_ = 0.04). At the same time, they were rarely nominated as a bully (*M*_intercept_ = 0.39*; M*_slope_ = 0.14*; M*_qslope_ = −0.01). The fourth joint trajectory (*N* = 722), labeled as the *uninvolved* trajectory, represents adolescents who were involved neither in bullying (*M*_intercept_ = 0.15; *M*_slope_ = 0.04; *M*_qslope_ = 0.00) nor in victimization (*M*_intercept_ = 0.14*; M*_slope_ = −0.03; *M*_qslope_ = 0.01) in the first two years of secondary education. In sum, of the four joint bullying and victimization trajectories, two were stable (high bully and uninvolved) and two were decreasing (decreasing victim and decreasing bully).

### Joint Trajectories and Their Social Position

Next, it was tested how different joint bullying and victimization trajectories related to adolescents’ social position in the first two years of secondary education. Table 5 displays the differences between joint trajectories on social position. Differences between trajectories in intercept at T1 and T6 as well as differences in slopes were assessed. With these results it could be determined whether someone’s social position has been determined already at the start of secondary education or whether belonging to a trajectory relates to changes in social position. Differences in intercepts and slopes were significant only when 95% *CI*s of means of a trajectory did not include the mean of the other trajectory and vice versa (Pfister and Janczyk 2013).

**Table 5:** Results multigroup analyses: Mean differences and 95% confidence intervals of social position per combined trajectory

Variable	Decreasing bully [95% CI]	High bully [95% CI]	Decreasing victim [95% CI]	Uninvolved [95% CI]
Social position T1 (intercept)				
Perceived popularity	2.09 [0.31; 3.86]^c^	3.52 [1.54; 5.50]^c^	−12.85 [−16.67; −9.03]^a^	−0.08 [−0.54; 0.38]^b^
Dislike	3.87 [2.71; 5.04]^b^	3.37 [2.52; 4.22]^b^	10.24 [7.26; 13.22]^c^	1.44 [1.09; 1.78]^a^
Like	8.35 [7.17; 9.53]^b^	9.22 [7.42; 11.02]^b^	3.92 [3.16; 4.68]^a^	9.40 [8.40; 10.40]^b^
Best Friends	5.13 [4.25; 6.01]^b^	4.26 [3.28; 5.24]^b^	0.91 [0.23; 1.60]^a^	5.22 [4.77; 5.66]^b^
Social position T6 (intercept)				
Perceived popularity	2.25 [0.96; 3.53]^c^	3.35 [2.19; 4.51]^c^	−10.02 [−13.29; −6.74]^a^	−0.24 [−0.68; 0.20]^b^
Dislike	3.10 [2.22; 3.98]^b^	3.99 [3.05; 4.93]^b^	6.03 [4.65; 7.42]^c^	1.92 [1.58; 2.25]^a^
Like	6.73 [5.40; 8.06]^b^	7.37 [5.44; 9.29]^b^	4.41 [3.07; 5.75]^a^	8.22 [7.33; 9.10]^b^
Best Friends	4.19 [3.27; 5.11]^b^	4.21 [3.20; 5.21]^b^	1.86 [0.89; 2.83]^a^	4.77 [4.39; 5.15]^b^
Social position (linear slope)				
Perceived popularity	0.71 [−0.64; 2.05]^a^	−0.02 [−1.46; 1.41]^a^	−0.60 [−3.10; 1.91]^a^	0.10 [−0.26; 0.47]^a^
Dislike	−0.70 [−1.40; 0.01]	0.86 [0.04; 1.67]	−0.34 [−2.24; 1.57]	0.25 [0.12; 0.38]
Like	−0.31 [−1.11; 0.50]^a^	−0.54 [−1.66; 0.59]^a^	0.37 [−0.50; 1.24]^a^	0.36 [0.12; 0.60]^a^
Best Friends	0.02 [−0.63; 0.68]^a^	0.33 [−0.38; 1.04]^a^	−0.06 [−0.63; 0.51]^a^	0.35 [0.19; 0.52]^a^

*CI* confidence interval, ^abc^indication of significant differences between means (based on Pfister and Janczyk 2013), with ^a^being the lowest value and ^c^being the highest value. *T* time point

**Fig. 2:**
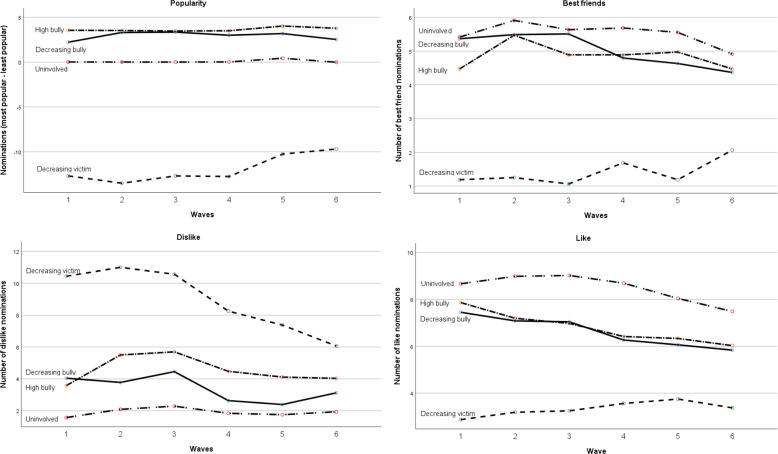
Descriptives popularity, friendship, dislike and like per estimated trajectory

As Fig. 2 displays, differences in social position were already visible at T1 and remained visible across the first two years of secondary education. This means that the relative social position for the groups did not change from T1 to T6. The next results therefore apply to both T1 (at the start of secondary education) and T6 (after two years of secondary education). First, both *bully groups* were clearly perceived as more popular than all other groups. Also, both bully groups as well as the uninvolved group were most liked and had most friends. Thus, both bully groups were able to attain both status and affection. However, both bully groups were more often nominated as being disliked in comparison to the uninvolved group. Second, the *uninvolved group* was least disliked, most liked, and had most friends. However, they were perceived as less popular in comparison with both bully groups. Third, the *decreasing victim group* was perceived as least popular, most disliked, least liked, and had the least friendships.

No significant differences between joint trajectories in slopes were found for perceived popularity, like, and best friend nominations. This means that belonging to a trajectory did not lead to changes over time in popularity, being liked, and the number of friends. However, there were significant differences between trajectories in the slopes of dislike nominations. While the decreasing bully group became less disliked over time (*M*_slope_ = −0.70), the uninvolved group (*M*_slope_ = 0.25) and, particularly, the high bully group (*M*_slope_ = 0.86) became more disliked over time (significant difference with decreasing bully group). Thus, when adolescents became less and less involved in bullying, they were decreasingly disliked while continuing to bully related to an increase in dislike nominations.

## Discussion

At the start of a new school, adolescents’ focal goal is to attain a strong social position in the peer group. A potential way to attain a strong social position seems by bullying others. However, it remains unknown whether bullies succeed in attaining status and affection in the first years at a new school. Longitudinal data spanning the first and second year of secondary education were used to examine joint bullying and victimization trajectories in relation to their social position. Parallel process LGCA revealed four joint bullying and victimization trajectories: a stable high bully group, a decreasing bully group, a decreasing victim group and a stable uninvolved group. In line with the often cited idea that bullying peaks directly at the start of a new school and decreases thereafter (Pellegrini and Long 2002), only stable or decreasing trajectories were found. Interestingly, two bully groups were found, that started their bullying behavior directly at the start of secondary education. However, stable high bullies continued to bully for at least the first two years of secondary education, whereas most bullies (decreasing bullies) slowly stopped their bullying behavior. It might be that they had reached a strong social position and did not find bullying functional to their goals anymore.

Differences in social position for all groups, with regard to both social status and affection, were comparable for T1 (at the start of secondary school) and T6 (after two years of secondary school). This indicates that the relative social position of the different joint trajectories was already determined at the start of secondary education. Adolescents immediately try to attain their social position at the start of secondary education, sometimes by using harmful bullying behavior (Pellegrini and Long 2002). After that, the social position of adolescents seems ‘set’. These findings imply that programs that aim to prevent bullying and to foster positive group formation can only be successful when they are implemented immediately at the start of secondary education, otherwise group hierarchy seems already set.

Bullying seems functional behavior because both bully groups were clearly perceived as more popular than the other groups. Also, both bully groups as well as the uninvolved group were most accepted and had most friends. Apparently, bullies were able to fulfill both social status and affection in order to optimize their social well-being (see SPF-theory; Ormel et al. 1999). The findings also indicated that the effects of bullying on adolescents’ social position can last for a longer period, because no differences between stable high bullies and decreasing bullies were found with regard to perceived popularity, being accepted, and having friends. However, stable high bullies became increasingly more rejected over time while decreasing bullies became less rejected over time. Maybe because these bullies continue to bully marginalized peers (Peets and Hodges 2014). Thus, while bullying seems accepted by peers at the start of secondary education (Rodkin et al. 2006), after some time bullying seems not an optimal strategy to attain affection anymore (Garandeau and Lansu 2019). Anti-bullying interventions may account for the increasing lack of affection that may hinder bullies’ long-term social development, and therefore focus on other strategies to improve adolescents’ social position (e.g., Ellis et al. 2016).

Victims were unable to attain social status and affection, both at the start of secondary school and two years later. Besides being bullied and perceived as unpopular, they were rejected and had the lowest number of friendships. Interestingly, victims in this sample appeared to be the ‘easy targets’ and not the social ‘rivals’ (Veenstra et al. 2010), because their social position was already low at the start of secondary education. Compared with primary education there were fewer victims in secondary education (Hong and Espelage 2012), which also means that there are fewer peers in a similar position that potentially share their plight (Huitsing et al. 2019). The lack of positive associating with peers may lead to persistent victimization, which might have important implications for victims’ (long term) mental health (Kaufman et al. 2018).

Finally, adolescents classified as uninvolved in bullying and victimization were least rejected, most accepted, and had most friends. However, they were perceived as less popular in comparison with both bully groups. Thus, uninvolved adolescents were able to attain affection, but not as much social status as bullies. It is possible that uninvolved adolescents do not value social status, and therefore strategically refrained from bullying and other forms of aggression. Although they were not involved as a bully or a victim, it is possible that they had a role in bullying situations as a bystander or defender, and therefore uninvolved adolescents can play an important role in reducing bullying by supporting victims (Salmivalli 2014).

This study had two important strengths. First, this study made use of longitudinal data in secondary education to relate bullying and victimization to adolescents’ social position. Second, peer-reports on relational information rather than self-reports were used, in order to provide a more reliable representation of bullying, victimization, and social position in adolescence. Self-reports often cause an underrepresentation of involvement in bullying and various other forms of aggression (Pepler et al. 2008). However, bully reports also have limitations. They especially may have caused an underrepresentation of victims in our sample, because bullies may be reluctant to nominate the victims they targeted.

This study also had a few limitations. First, the analytical approach could have benefited from information on how much adolescents valued social status or affection. The prioritizing of social status may differ between adolescents and change across time (Malamut et al. 2020). For example, it is possible that some adolescents do not seek status and are fine with having just a few friends, and therefore in their opinion reached the desired levels of social status and affection. Second, although this study made use of a large sample, all students came from two schools located in the middle and north of the Netherlands. There were no indications that the peer processes at these schools differ with the processes in other Dutch schools, but it is still possible that cohort or regional differences may affect the prevalence estimates of the behavior under investigation. Third, the pair-wise group comparisons after the parallel process LGCA were performed at the group level precluding to estimate individual differences between adolescents within the groups. Future research might focus more on within-person changes across time instead of focusing on group comparisons.

## Conclusion

At the start of secondary education, adolescents are eager to attain a strong social position in their new school class. A strategy to attain a strong social position is by bullying others. However, it remains unknown if bullies succeed in the end. Therefore, in this study, bullying and victimization trajectories were related to adolescents’ social status and affection, using longitudinal data spanning the first years of secondary education. Results indicated that bullies were able to attain both social status and affection. Uninvolved adolescents were able to attain affection, but less social status and victims attained neither status nor affection. Furthermore, the relative social position of the different joint trajectories was already determined at the start of secondary education and did not change over time. This has important implications for bullying prevention in secondary schools. Programs that aim to prevent bullying and to foster positive group formation in adolescence are likely to only be successful when they are implemented immediately at the start of secondary education. We also found that adolescents who did not only bully at the start of secondary education but also in the next years became, although popular, increasingly rejected. The increase of rejection may hinder bullies’ long-term social development. In that way, adolescents may benefit from interventions that link social status and affection to prosocial behavior.

## Appendix 1

Flow diagram showing participant in- and exclusion
